# β-amyloid deposition is shifted to the vasculature and memory impairment is exacerbated when hyperhomocysteinemia is induced in APP/PS1 transgenic mice

**DOI:** 10.1186/alzrt262

**Published:** 2014-06-09

**Authors:** Tiffany L Sudduth, Erica M Weekman, Holly M Brothers, Kaitlyn Braun, Donna M Wilcock

**Affiliations:** 1 Department of Physiology, Sanders-Brown Center on Aging, University of Kentucky, 800 S. Limestone St., Lexington, KY 40536, USA

## Abstract

**Introduction:**

Vascular dementia is the second most common cause of dementia after Alzheimer’s disease (AD). In addition, it is estimated that almost half of all AD patients have significant cerebrovascular disease comorbid with their AD pathology. We hypothesized that cerebrovascular disease significantly impacts AD pathological progression.

**Methods:**

We used a dietary model of cerebrovascular disease that relies on the induction of hyperhomocysteinemia (HHcy). HHcy is a significant clinical risk factor for stroke, cardiovascular disease and type 2 diabetes. In the present study, we induced HHcy in APP/PS1 transgenic mice.

**Results:**

While total β-amyloid (Aβ) load is unchanged across groups, Congophilic amyloid deposition was decreased in the parenchyma and significantly increased in the vasculature as cerebral amyloid angiopathy (CAA; vascular amyloid deposition) in HHcy APP/PS1 mice. We also found that HHcy induced more microhemorrhages in the APP/PS1 mice than in the wild-type mice and that it switched the neuroinflammatory phenotype from an M2a biased state to an M1 biased state. Associated with these changes was an induction of the matrix metalloproteinase protein 2 (MMP2) and MMP9 systems. Interestingly, after 6 months of HHcy, the APP/PS1 mice were cognitively worse than wild-type HHcy mice or APP/PS1 mice, indicative of an additive effect of the cerebrovascular pathology and amyloid deposition.

**Conclusions:**

These data show that cerebrovascular disease can significantly impact Aβ distribution in the brain, favoring vascular deposition. We predict that the presence of cerebrovascular disease with AD will have a significant impact on AD progression and the efficacy of therapeutics.

## Introduction

*Vascular dementia* (VaD) is a term that encompasses many different causes of vascular disease and dysfunction in the brain that can lead to cognitive impairment [1]. Included in this assortment are stroke, aneurysm, damage due to chronic hypoperfusion and damage due to chronic hypertension. VaD is the second most common cause of dementia behind Alzheimer’s disease (AD). Furthermore, it is estimated that approximately 40% of AD patients also have cerebrovascular disease as a comorbidity [2-4]. It is likely that the presence of cerebrovascular disease independently contributes to the clinical symptoms of dementia.

We recently showed that induction of hyperhomocysteinemia in wild-type (WT) mice results in cognitive deficits, neuroinflammation and cortical microhemorrhages [5]. Homocysteine is a non-protein-forming sulfur amino acid involved in methylation and transsulfuration [6]. It is the product of the methylation cycle and also a substrate for cysteine biosynthesis. Elevated levels of homocysteine, termed *hyperhomocysteinemia* (HHcy), is considered a risk factor for cardiovascular and cerebrovascular diseases [7]. HHcy itself is sufficient to induce cognitive deficits in both rat and mouse models [8,9]. In mice, HHcy is induced by administering a diet deficient in vitamins B6 and B12 as well as folate, and supplemented with methionine. This diet then drives the metabolic pathway to produce HHcy with minimal breakdown into cysteine.

In the present study, we hypothesized that cerebrovascular disease would significantly alter AD pathological progression. To test this possibility, we administered a diet to induce HHcy in APP/PS1 transgenic mice. We administered the diet for 6 months, beginning when the mice were 6 months of age. Researchers in previously published studies using models of HHcy in APP transgenic mice have focused on total Aβ changes and have reported both no change in Aβ [10,11] and also modest increases in Aβ [12,13]. Interestingly, the investigators in these studies did not examine cerebrovascular changes or neuroinflammation. In our present study, we found that amyloid-β (Aβ) levels were not affected by HHcy, but we observed that the deposited amyloid was now more associated with the vasculature. We noted an altered neuroinflammatory response and an additive effect of HHcy on spatial memory impairment in APP/PS1 mice.

## Materials and methods

### Animals

Forty APP/PS1 mice [14], age 6 months, were placed on either a diet with low levels of folate, vitamins B6 and B12 and enriched with methionine (HHcy study group; *n* = 20, comprising 12 females and 8 males) or a control diet that nutritionally matched the experimental diet with normal levels of methionine, folate and vitamins B6 and B12 (control group; *n* = 20, comprising 12 females and 8 males). Additionally, 32 WT mice (C57BL/6 littermate controls from the breeding of the APP/PS1 mice), age 6 months, were placed on either the HHcy diet (*n* = 16, comprising 8 females and 8 males) or the control diet (*n* = 16, comprising 8 females and 8 males). The HHcy diet was Harlan Teklad TD97345, and the control diet was Harlan Teklad 5001 C (both from Harlan Laboratories, Madison, WI, USA). All mice received the diet for 6 months. Mice were weighed weekly to ensure that no significant malnourishment due to the diet was occurring. The study was approved by the University of Kentucky Institutional Animal Care and Use Committee and conformed to the National Institutes of Health’s *Guide for the Care and Use of Laboratory Animals*. For all analyses, sex differences were analyzed and none were noted, so males and females were combined.

### Behavior testing

Radial arm water maze testing was performed at the University of Kentucky Rodent Behavior Core. The 2-day radial arm water maze protocol was carried out as previously published [15]. Briefly, a six-arm maze was submerged in a pool of water, and a platform was placed at the end of one arm (equipment and tracking software from Noldus Information Technology, Leesburg VA, USA). Each mouse was subjected to 15 trials per day for 2 days. Each mouse began each trial in a different arm while the arm containing the platform remained the same. The number of errors (incorrect arm entries) was counted over a 1-minute period. The errors were averaged over three trials, resulting in ten blocks for the 2-day period: Day 1 comprised blocks 1 to 5, and day 2 comprised blocks 6 to 10.

### Tissue processing and histology

After the mice were injected with a lethal dose of pentobarbital, we collected their blood for plasma, and the mice were perfused intracardially with 25 ml of normal saline. The brains were rapidly removed and bisected in the midsagittal plane. The left half was immersion-fixed in 4% paraformaldehyde for 24 hours, and the right half was dissected into anterior cerebral cortex, posterior cerebral cortex, striatum, hippocampus, thalamus, cerebellum and rest of brain. The posterior cerebral cortex and rest of brain were combined and immediately homogenized in phosphate-buffered saline (PBS) for zymography (see detailed methodology below). The remaining pieces were flash-frozen in liquid nitrogen and stored at −80°C. The left hemibrain was passed through a series of 10%, 20% and 30% sucrose solutions for cryoprotection, and then 25-μm frozen horizontal sections were collected serially with a sliding microtome and afterward stored floating in PBS containing sodium azide at 4°C. Plasma was analyzed by a veterinary reference diagnostics service for lipid and homocysteine levels (ANTECH Diagnostics, Fishers, IN, USA).

Eight sections equally spaced 600 μ apart were selected for free-floating immunohistochemistry for total Aβ (1:3,000 dilution rabbit polyclonal antibody; Invitrogen, Carlsbad, CA, USA) and CD45 (1:3,000 dilution rat monoclonal antibody; Thermo Scientific, Rockford, IL, USA). The method used for free-floating immunohistochemistry has been described previously [16]. Sixteen sections equally spaced 300 μ apart were mounted on slides and stained for Prussian blue as described previously [17].

### Enzyme-linked immunosorbent assay measurement

Protein was extracted for Aβ analysis from the right frontal cortex using a two-step extraction method. First, the brain was homogenized in PBS containing a complete protease and phosphatase inhibitor (Pierce Biotechnology, Rockford, IL, USA). These samples were centrifuged at 16,000 × *g* at 4°C for 1 hour. The supernatant was removed and became the “soluble” extract. The resulting pellet was homogenized in 100 μl of 70% formic acid and centrifuged again at 16,000 × *g* at 4°C for 1 hour. The supernatant was removed and neutralized 1:20 with 1 M Tris-HCl and became the “insoluble” extract. The protein concentration for both the soluble and insoluble extracts was determined using a bicinchoninic acid (BCA) protein assay according to the manufacturer’s instructions (Thermo Scientific). We used the Meso Scale Discovery multiplex enzyme-linked immunosorbent assay (ELISA) system to measure Aβ38, Aβ40 and Aβ42 (Meso Scale Discovery, Rockville, MD, USA). The ELISA experiments were run according to the kit manufacturer’s instructions.

### Quantitative real-time RT-PCR

RNA was extracted from the frozen right hippocampus using the TRIzol Plus RNA Purification Kit (Life Technologies, Grand Island, NY, USA) according to the manufacturer’s instructions. RNA was quantified using a NanoDrop spectrophotometer (Thermo Scientific), and cDNA was produced using a High Capacity cDNA kit (Life Technologies) according to the manufacturer’s instructions. Real-time RT-PCR was performed using a TaqMan gene expression assay kit (Life Technologies) according to the manufacturer’s instructions and as previously described [18]. All genes were normalized to 18S ribosomal RNA. We determined fold changes using the δ method for mice that were fed the experimental diet compared to mice given the control diet [19].

### Gelatin zymography

The enzymatic activities of tissue matrix metalloproteinases were measured by performing gelatin zymography in brain samples. Protein was extracted from fresh brain tissue in PBS, specifically the right posterior cerebral cortex and midbrain together, and quantified immediately using a BCA protein assay kit (Pierce Biotechnology) according to the manufacturer’s instructions. Protein samples were immediately separated on a precast 10% gelatin zymogram gel (Life Technologies). The gel was removed, incubated in zymogram renaturing buffer for 30 minutes, equilibrated for 30 minutes in zymogram developing buffer at room temperature and then incubated overnight at 37°C with gentle agitation in fresh zymogram developing buffer (all buffers obtained from Life Technologies). The next day, the gel was washed gently with water and incubated in IRDye blue protein stain, a Coomassie blue stain (LI-COR Biosciences, Lincoln, NE, USA). The gel was stained for 1 hour and then destained in H_2_O until clear band resolution was apparent (approximately 1 hour). The gel was scanned on an Odyssey imager, and semiquantitative densitometric analysis was performed using Odyssey imaging software (LI-COR Biosciences).

### Analysis

Data are presented as mean ± SEM. Statistical analysis was performed using the JMP statistical analysis software program (SAS Institute, Cary, NC, USA). Radial arm water maze data and rotarod performance were analyzed by repeated-measures analysis of variance (ANOVA) to assess the overall effect of diet. For the radial arm water maze data, we also performed Student’s *t*-test on individual block data. For gene expression analysis, we used the Mann–Whitney–Wilcoxon test. For other data, one-way ANOVA and Student’s *t*-test were performed. Statistical significance was assigned when the *P*-value was <0.05.

## Results

Administration of the diet deficient in B6, folate and B12 and enriched in methionine for 6 months, from 6 to 12 months of age, resulted in significant elevations in plasma homocysteine levels in both WT and APP/PS1 transgenic mice (WT control: 6.85 ± 0.8 μmol/L, WT HHcy: 68.23 ± 12.1 μmol/L, APP/PS1 control: 7.65 ± 1.4 μmol/L and APP/PS1 HHcy: 64.21 ± 8.3 μmol/L). There was no significant difference between WT and APP/PS1 transgenic mice in the plasma homocysteine levels induced by the diet. In C57BL/6 mice, normal homocysteine levels are considered to be 5 to 12 μmol/L. Hyperhomocysteinemia can be categorized as mild (12 to 30 μmol/L), moderate (30 to 100 μmol/L) or severe (>100 μmol/L) [20]. On the basis of the levels we observed in our experimental mice, HHcy levels were moderate. None of our mice reached the plasma level of homocysteine classified as severe HHcy.

**Figure 1:**
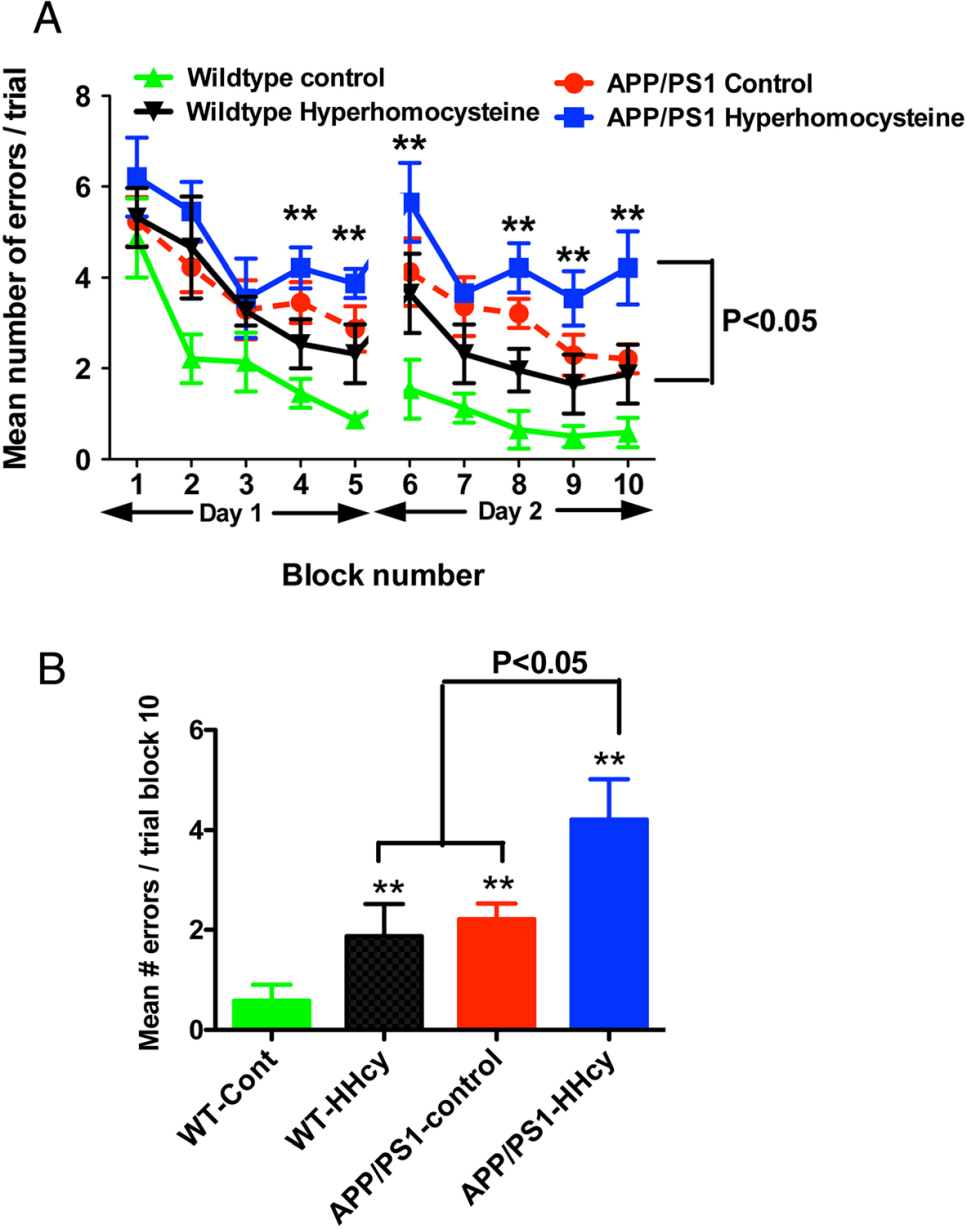
**Cognitive deficits are additive when hyperhomocysteinemia is induced in APP/PS1 transgenic mice. (A)** Two-day radial arm water maze data are graphed. The mean number of errors per trial were calculated for blocks 1 to 10 (each block comprised three trials). Asterisks indicate significant differences for hyperhomocysteinemia (HHcy) wild-type (WT) mice (*n* = 16), APP/PS1 control mice (*n* = 20) and HHcy APP/PS1 mice (*n* = 20) compared to WT control mice (*n* = 16). **P < 0.01. **(B)** Final block data only are graphed (block 10). Asterisks indicate significance compared to WT controls. **P < 0.01.

In the 2-day radial arm water maze task, WT mice began the test naïve and made an average of five errors per trail (Figure 1). The WT control mice were making less than one error per trail by the end of the first day, and this performance continued into day 2. HHcy WT mice were impaired in performing the task and were significantly impaired compared to WT control mice throughout the second day of testing (*P* < 0.01) (Figure 1A). APP/PS1 transgenic mice fed the control diet showed a level of impairment similar to that observed in the HHcy WT mice (*P* < 0.01) (Figure 1A). Interestingly, HHcy APP/PS1 transgenic mice showed more severe impairment, with an apparent additive effect of HHcy combined with the APP/PS1 transgene (*P* < 0.01) (Figure 1A). When we plotted the mean number of errors per trial for the final block of day 2, we found that both the HHcy WT mice and the APP/PS1 mice were making a mean of approximately 1.8 errors per trial, whereas the HHcy APP/PS1 mice were making a mean of around 4.0 errors per trial (*P* < 0.01) (Figure 1B).

**Figure 2:**
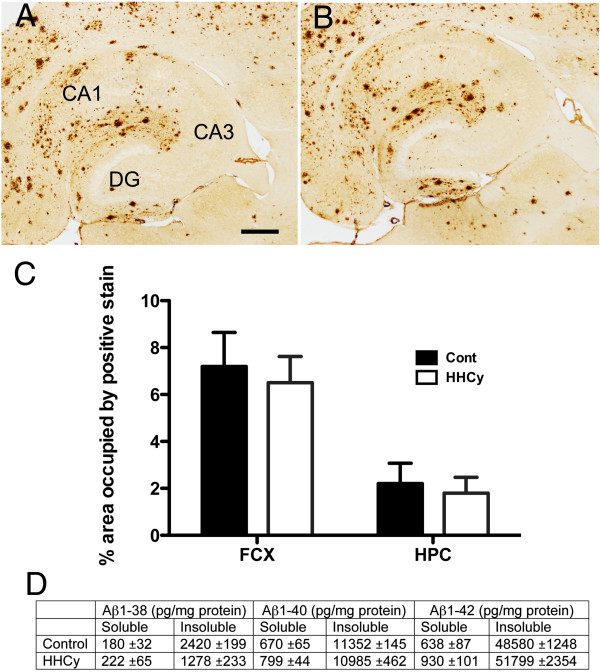
**Total β-amyloid is unaltered by hyperhomocysteinemia in APP/PS1 transgenic mice.** Total β-amyloid (Aβ) immunohistochemistry in the hippocampus of APP/PS1 mice on either the control diet **(A)** or the hyperhomocysteinemia (HHcy) diet **(B)**. (A) shows the CA1, CA3 and dentate gyrus (DG) for orientation. Original magnification = 40×. Scale = 120 μm. **(C)** Quantification of percent area occupied by positive staining for Aβ in the hippocampus (HPC) and frontal cortex (FCX) of APP/PS1 transgenic mice fed either the control diet (*n* = 20, black bars) or the HHcy diet (*n* = 20, white bars). Error bars show SEM. **(D)** Biochemical quantification of Aβ_1–38_, Aβ_1–40_ and Aβ_1–42_ in both the soluble and insoluble protein extracts ± SEM.

To determine whether HHcy had resulted in Aβ changes in the APP/PS1 mice, we first assessed total Aβ by immunohistochemistry. The control APP/PS1 transgenic mice showed a typical staining pattern for the 12-month age point in both the hippocampus (Figure 2A) and the frontal cortex. However, no change in total Aβ staining was observed in HHcy APP/PS1 mice (Figure 2B). Quantitatively, total Aβ staining was unchanged with HHcy in APP/PS1 mice in either the frontal cortex or the hippocampus (Figure 2C). To confirm that no changes in Aβ production were occurring, we performed biochemical analysis of brain tissue using an Aβ triplex ELISA kit that measures Aβ_1–38_, Aβ_1–40_ and Aβ_1–42_ (Meso Scale Discovery). In both the soluble and insoluble fractions of the cerebral cortex, we found no significant differences in any of the Aβ species measured between the control APP/PS1 and HHcy APP/PS1 mice (Figure 2D).

**Figure 3:**
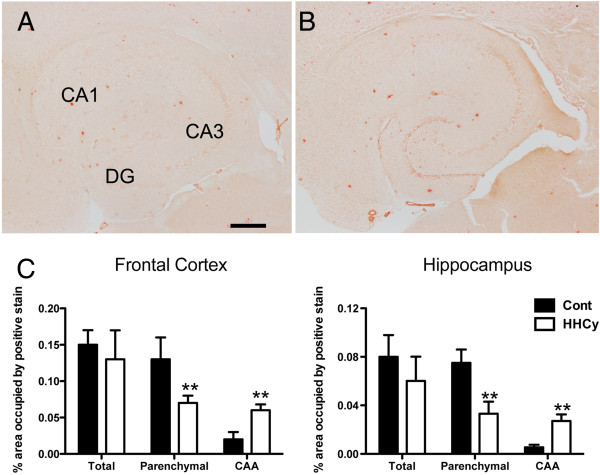
**Amyloid is redistributed to the vasculature in the hyperhomocysteinemia APP/PS1 transgenic mice.** Congo red staining in the hippocampi of APP/PS1 mice fed the control diet **(A)** or the hyperhomocysteinemia (HHcy) diet **(B)**. **(A)** shows the CA1, CA3 and dentate gyrus (DG) for orientation. Scale bar = 120 μm for **(A)** and **(B)**. **(C)** Quantification of percent area occupied by positive Congo red staining in the hippocampus and frontal cortex of APP/PS1 transgenic mice on either control (Cont) diet (*n* = 20, black bars) or the HHcy diet (*n* = 20, white bars). Each graph shows total Congo red, parenchymal Congo red and cerebral amyloid angiopathy (CAA) Congo red. **P < 0.01 compared to APP/PS1 control. Error bars show SEM.

We specifically examined the compact amyloid deposits by Congo red staining. Again, for the 12-month-old control APP/PS1 mice, we observed a typical staining pattern in the hippocampus (Figure 3A) and the frontal cortex. What was striking is that we saw showed more Congo red staining of the cerebrovasculature in the HHcy APP/PS1 mice (Figure 3B). We quantified this difference using a previously published method of distinguishing between cerebral amyloid angiopathy (CAA) and parenchymal amyloid [21]. We thus found that, whereas total Congo red levels of the control APP/PS1 mice and HHcy APP/PS1 mice were not different, HHcy APP/PS1 mice showed fewer parenchymal amyloid deposits and increased CAA levels relative to the control APP/PS1 mice. In both the frontal cortex and hippocampus, we found a 50% to 60% reduction in parenchymal amyloid and over double the amount of CAA in the HHcy APP/PS1 mice relative to the control APP/PS1 mice (*P* < 0.01) (Figure 3C).

**Figure 4:**
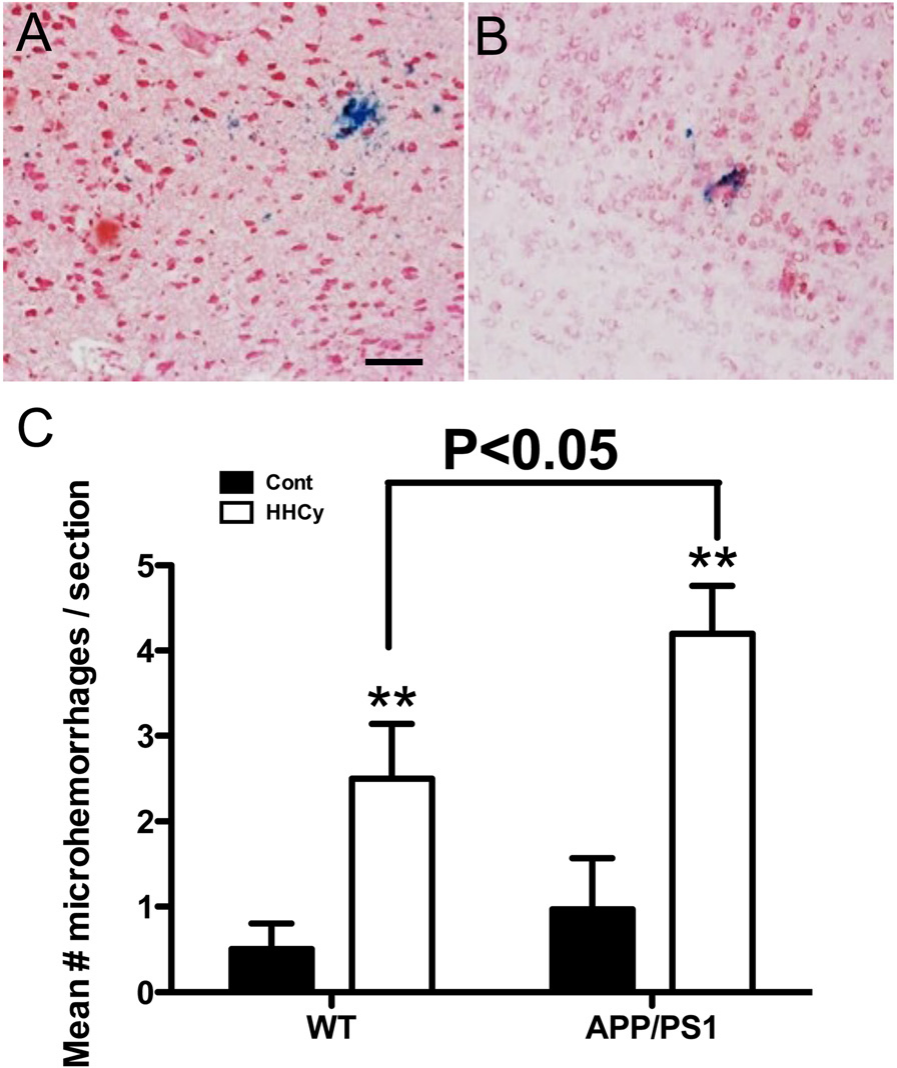
**Microhemorrhages are increased by hyperhomocysteinemia in both wild-type and APP/PS1 transgenic mice. (A)** and **(B)** Prussian blue–positive microhemorrhages in the cerebral cortices of APP/PS1 mice fed the hyperhomocysteinemia (HHcy) diet. Both images were obtained at 200× original magnification with a neutral red background stain. Scale bar = 25 μm. **(C)** Mean number of microhemorrhages per section for wild-type (WT) and APP/PS1 transgenic mice fed the control (*n* = 16 for WT mice and *n* = 20 for APP/PS1 mice, black bars) and the HHcy diet (*n* = 16 for WT mice and *n* = 20 for APP/PS1 mice, white bars). **P < 0.01 compared to the control group for the given genotype. Error bars show SEM.

HHcy results in significant induction of microhemorrhage in WT mice [5]. Prussian blue histology was used to assess microhemorrhage levels. We found that both the WT and control APP/PS1 mice typically showed few microhemorrhages, with less than one Prussian blue–positive profile per section. However, HHcy induced significant microhemorrhages in both the WT and APP/PS1 mice (*P* < 0.01) (Figures 4A and 4B). HHcy induced a threefold increase in microhemorrhages in the WT mice and a fourfold increase in microhemorrhages in the APP/PS1 mice (Figure 4C). Importantly, the APP/PS1 mice responded to HHcy with significantly more microhemorrhages than we saw in the WT mice (*P* < 0.05) (Figure 4C).

**Figure 5:**
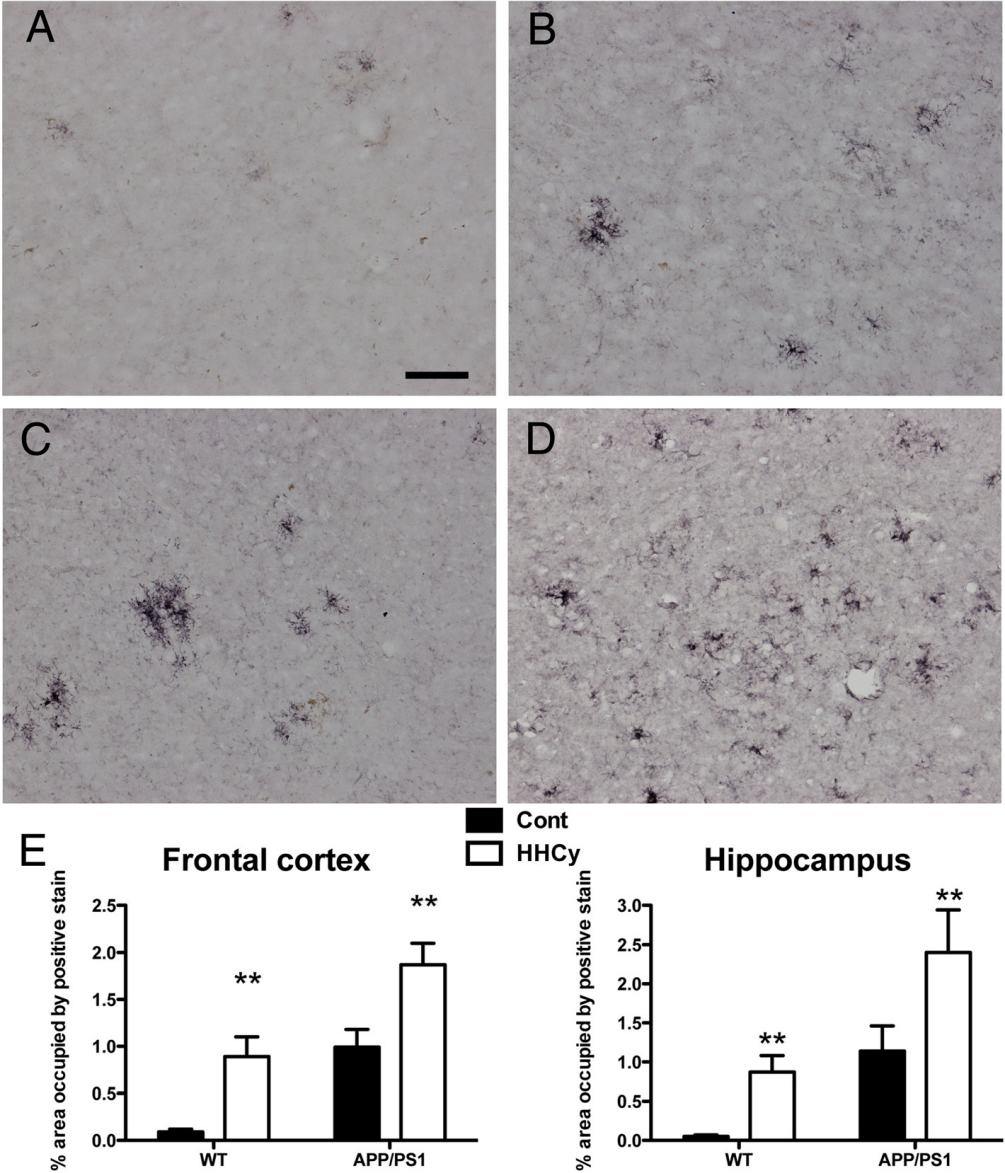
**CD45 expression by microglial cells is increased by hyperhomocysteinemia in both wild-type and APP/PS1 mice.** CD45 immunohistochemical staining in the frontal cortices of wild-type (WT) mice fed the control diet **(A)** or the hyperhomocysteinemia (HHcy) diet **(B)** and APP/PS1 mice fed the control diet **(C)** or the HHcy diet **(D)**. Original magnification = 200×. Scale bar = 25 μm. **(E)** Quantification of percent area occupied by positive CD45 immunoreactivity in the frontal cortex and hippocampus of WT and APP/PS1 mice on both control (*n* = 16 for WT and *n* = 20 for APP/PS1, black bars) and HHcy diet (*n* = 16 for WT and *n* = 20 for APP/PS1, white bars). **P < 0.01 compared to the control group for a given genotype. Error bars show SEM.

Neuroinflammation is induced by HHcy in WT mice [5]. CD45 immunohistochemistry revealed enhanced microglial activation with HHcy in both the WT and APP/PS1 mice (Figure 5). We found that WT controls had very little CD45 expression in the brain (Figures 5A and 5E), but that, with HHcy, WT mice showed microglial activation associated with the cerebrovasculature (Figures 5B and 5E). Control APP/PS1 mice showed microglial activation associated with the amyloid deposits in the brain (Figures 5C and 5E), but, following HHcy induction, APP/PS1 mice showed significantly greater CD45 expression, particularly in association with the cerebrovasculature (Figures 5D and 5E).

**Figure 6:**
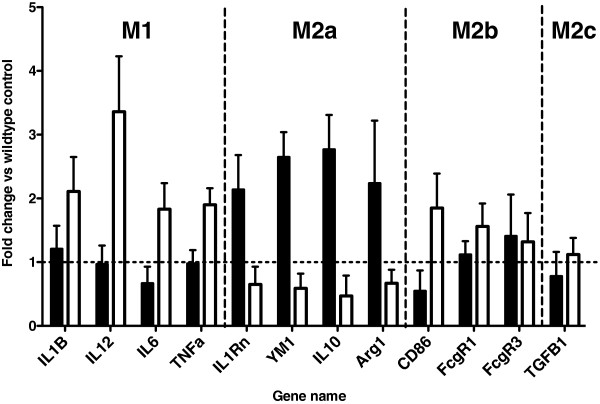
**Hyperhomocysteinemia induces an inflammatory phenotype shift in the APP/PS1 transgenic mice away from M2a and toward M1.** The graph shows fold changes for each gene relative to the wild-type control mice. The dashed line at 1 indicates the normal wild-type expression of these genes. IL, Interleukin; TNF, Tumor necrosis factor; IL1rn, Interleukin 1 receptor antagonist; Arg1, Arginase type 1; TGF, Transforming growth factor; FcgR1, Fc gamma receptor 1 *P < 0.05, **P < 0.01 compared to APP/PS1 control (*n* = 16 for each wild-type group and *n* = 20 for each APP/PS1 group). Error bars show SEM.

We further examined the neuroinflammatory response in the present study by using the macrophage phenotype categories M1, M2a, M2b and M2c [22,23]. HHcy results in an M1-type neuroinflammation in WT mice [5]. We have also previously shown that amyloid-depositing Tg2576 mice show an M2a phenotype when amyloid has accumulated [18]. In the present study, we show that HHcy resulted in increased expression of the M1 markers interleukin 1β (IL-1β), tumor necrosis factor α, IL-12 and IL-6 in the APP/PS1 mice (Figure 6). Together with this increase in M1 markers, we observed a concomitant decrease in the M2a markers IL-1- receptor antagonist, IL-10, Ym1 and Arg1 (Figure 6). The M2b and M2c phenotypes were largely unaffected in both the control APP/PS1 and HHcy APP/PS1 mice (Figure 6).

**Figure 7:**
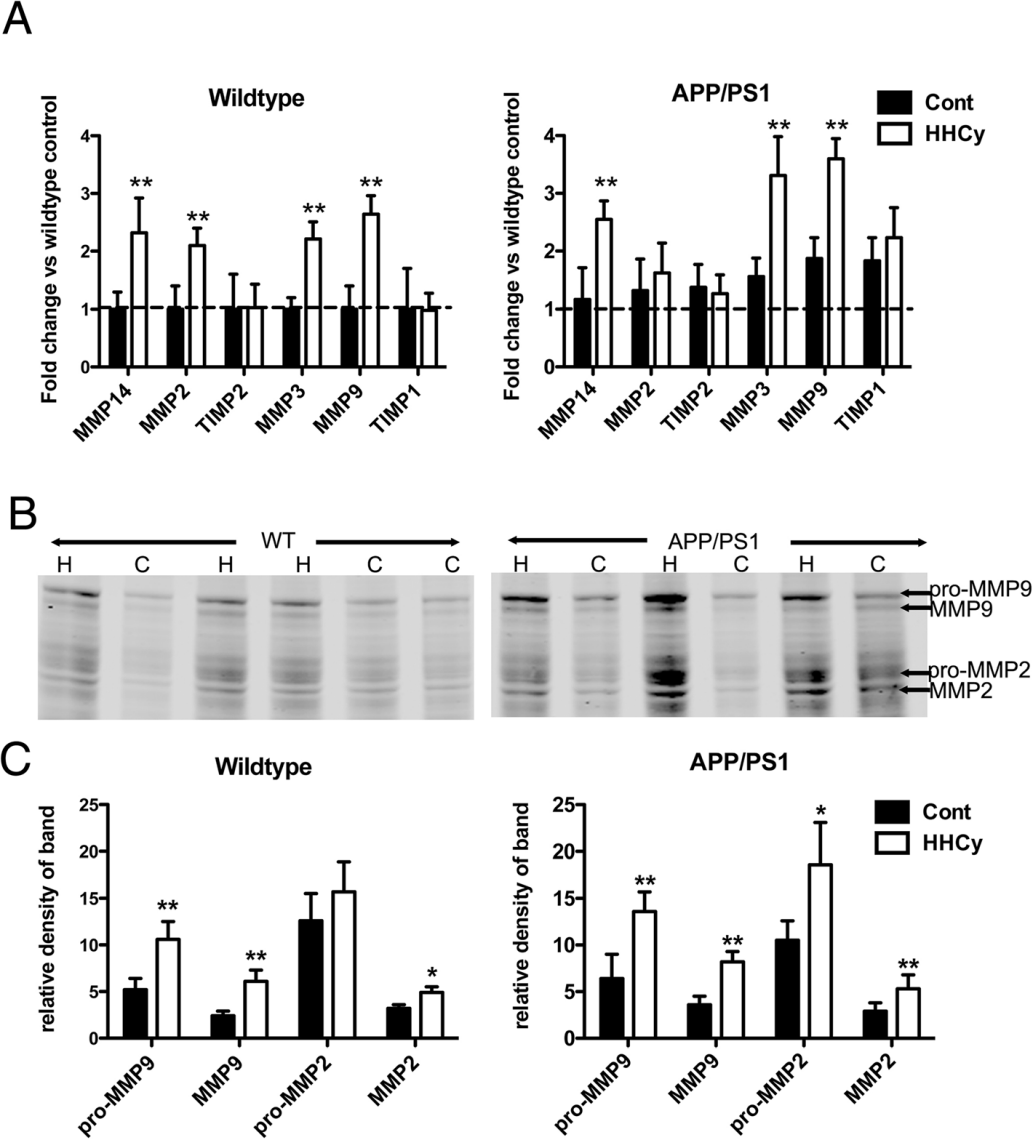
**Matrix metalloproteinases 2 and 9 are activated by hyperhomocysteinemia in wild-type and APP/PS1 transgenic mice. (A)** Graphed quantitative RT-PCR results for components of the matrix metalloproteinases 2 and 9 (MMP2 and MMP9) systems in both the wild-type (WT) and APP/PS1 mice. TIMP, Tissue inhibitor of metalloproteinase. **P < 0.01 compared to control (Cont) for the given genotype. **(B)** Gelatin zymogram color-inverted to highlight the digested bands in black. As indicated to the right, on the basis of molecular weight, we can determine that the bands highlighted correspond to pro-MMP9, MMP9, pro-MMP2 and MMP2. H, Hyperhomocysteinemia (HHcy) mice; C, control mice. **(C)** Quantification of band density for bands of interest highlighted in (B) for wild-type and control mice. *P < 0.05, **P < 0.01 compared to the control group for the given genotype (*n* = 16 for each wild-type group and *n* = 20 for each APP/PS1 group). Error bars show SEM.

It is well known that matrix metalloproteinase (MMP) activation, in particular MMP2 and MMP9, is a critical mediator of hemorrhagic transformation after aneurysm formation and stroke [24,25]. We have previously shown that MMPs are activated by anti-Aβ immunotherapy in studies involving microhemorrhage induction [26], and we have also shown this activation in WT HHcy mice [5]. Here we show that gene expression of MMP2 and MMP9 system components are increased, relative to WT controls, in both the WT and APP/PS1 mice with induced HHcy (Figure 7A). In addition, we have found, by gelatin zymography, increased activity of MMP2 and MMP9 in both HHcy WT and HHcy APP/PS1 mice (Figures 7B and 7C). Interestingly, MMP9 activation was particularly increased in the HHcy APP/PS1 mice (relative intensity = 8.2), which is significantly greater than that measured in the HHcy WT mice (relative intensity = 6.1) (*P* < 0.05 for MMP9 activity and gene expression between HHcy WT and HHcy APP/PS1 mice).

## Discussion

Although VaD is the second most common cause of dementia after AD, it remains relatively understudied, with the challenge being a lack of animal models available to study the mechanisms by which cerebrovascular disease leads to dementia. We recently described a model in which cortical microhemorrhages, neuroinflammation and cognitive deficits develop when HHcy is induced in C57BL/6 mice, suggesting that the HHcy model is an appropriate model for the study of some forms of VaD. We have been particularly interested in the comorbidity of VaD and AD, given that it is estimated that around 40% of AD patients also have significant cerebrovascular disease pathology. To determine the effect of cerebrovascular disease on amyloid pathology, we induced HHcy in APP/PS1 transgenic mice. We found that the cognitive deficits are additive when HHcy is induced in APP/PS1 mice. Together with this additive effect, we found a shift of Aβ from the parenchyma to the vasculature in the absence of total Aβ changes. In parallel with increased vascular Aβ, HHcy induced microhemorrhages in both the WT and APP/PS1 mice, but significantly more microhemorrhages occurred in the HHcy APP/PS1 mice than in the HHcy WT mice. We saw a strong neuroinflammatory phenotype switch from an M2a biased state to an M1 biased state. We also observed an associated activation of MMP2 and MMP9 systems.

We have previously shown that HHcy can lead to cognitive deficits in WT mice [5]. In addition, in previous studies of HHcy, researchers have shown behavioral deficits in the Morris water maze test in mice [8] and in the radial arm maze test in rats [9]. In the present study, we show that WT mice fed the HHcy-inducing diet developed moderate cognitive deficits similar to those we have previously described. We have found that these deficits are indistinguishable from those of 12-month-old APP/PS1 transgenic mice fed a control diet. However, HHcy APP/PS1 mice showed significantly greater deficits in the radial arm water maze test compared to the HHcy WT and control APP/PS1 mice. In fact, when we examined only the mean number of errors per trial for the final block of testing, we found that the number of errors made by the HHcy APP/PS1 mice were double those made by the HHcy WT and the APP/PS1 control mice, suggesting an additive effect of the amyloid and cerebrovascular pathology.

The additive effect on cognition of HHcy in APP/PS1 mice is consistent with conclusions derived from a human imaging study published by the Alzheimer’s Disease Neuroimaging Initiative. These investigators suggested that cerebrovascular pathology acts independently of AD pathology, creating an additive effect on overall clinical dementia [27]. Also, in autopsy studies, researchers have shown that AD pathology is less severe in patients who have cerebrovascular disease than in AD patients with matching cognitive scores who do not have cerebrovascular disease [28]. Further, in a study with clinical outcome measures of dementia in a population with autopsy-confirmed diagnoses, the researchers found that mixed pathology (VaD and AD) shows essentially an additive effect on test scores for memory, nonverbal memory and executive function. Patients with mixed pathology performed more poorly than those with only AD or VaD [29].

Of particular interest in our present study is the apparent redistribution of Aβ deposition to the vasculature. Previous studies in which researchers induced HHcy in APP transgenic mice have produced mixed results. Some investigators have found modest increases in total Aβ levels [12,13], whereas others have discovered no difference [10,11], with multiple methodological approaches used across studies. As we show in Figure 2, total Aβ was unchanged with induction of HHcy. The most dramatic effect occurred when we separated out the parenchymal and cerebrovascular amyloid deposition stained with Congo red. With the use of our previously published method to separately quantify parenchymal amyloid and CAA [17,21], we found a significant decrease in parenchymal amyloid along with a significant concomitant increase in CAA.

In previous immunotherapy study [17], we found a similar redistribution of amyloid, albeit in the presence of an overall reduction in Aβ, in APP transgenic mice. In that study, we showed that anti-Aβ immunotherapy lowers total Aβ but causes a concomitant increase in CAA deposition. The common link between that study and our present HHcy study is the modulation of the neuroinflammatory phenotype. Both immunotherapy and HHcy shift the neuroinflammatory state away from an M2a biased phenotype to an M1 biased state [18]. The role of the neuroinflammatory phenotype or, more broadly, microglia in the regulation of amyloid deposition as CAA or parenchymal plaques is relatively unknown. The results of our present study further support the concept that modulation of the neuroinflammatory phenotype is associated with altered distribution of amyloid. Indeed, with immunotherapy, minimizing the interaction between the antibody and microglia through deglycosylation of the antibody also minimizes accumulation of amyloid at the vasculature while preserving the parenchymal amyloid-lowering properties of the antibody [30]. In addition, HHcy is known to influence vascular function directly [31-33]. It is possible that the perivascular drainage of Aβ, a major clearance pathway of Aβ from the brain [34], was affected in our HHcy mice. Low-density lipoprotein-related protein (LRP) is known to transport Aβ across the endothelium and out of the brain, and the receptor for advanced glycation end products (RAGEs) is known to transport Aβ across the endothelium into the brain. This transport system is also known to be crucial for the transport of Aβ across the blood–brain barrier [35,36]. HHcy has been shown to enhance the expression of RAGEs in the vasculature, which could impact the equilibrium of Aβ between the plasma and the brain [37]. In future studies, we will examine both perivascular transport and LRP-RAGE-mediated endothelial transport in the HHcy model.

Consistent with previous findings, in our present study, HHcy resulted in significant microhemorrhages in both the WT and APP/PS1 mice, although the APP/PS1 mice showed significantly more microhemorrhages than the WT mice. There also appears to have been greater induction of the MMP9 system in the HHcy APP/PS1 mice relative to the HHcy WT mice. Because MMP9 is heavily implicated in the induction of cerebrovascular degeneration, we hypothesize that this activation mediates microhemorrhage onset. MMP9 has been shown to be a critical mediator for hemorrhagic transformation following a cerebral ischemic event [25], white-matter lesion formation and blood–brain barrier (BBB) breakdown in VaD [38]. In addition, MMP2 and MMP9 levels have been shown to be associated with cerebral hemorrhage resulting from vascular amyloid deposition (that is, CAA) [39,40]. In our present study, by gelatin zymography, we investigated not only the expression of components of the MMP2 and MMP9 systems but also the activity of these metalloproteinases. Gelatin zymography confirmed that the activity levels of MMP2 and MMP9 were significantly increased by HHcy and that this increase was slightly greater in the APP/PS1 mice than in the WT mice.

## Conclusions

We have successfully modeled mixed dementia by combining the HHcy model of VaD with the amyloid-depositing APP/PS1 transgenic mouse. This novel approach shows enhanced CAA, altered neuroinflammatory profiles, activation of MMPs and, most importantly, an additive effect on cognitive outcomes similar to the additive effects seen in human studies.

## Abbreviations

AD: Alzheimer’s disease; APP: Amyloid precursor protein; Arg1: Arginase type 1; Aβ: Amyloid-β; CAA: Cerebral amyloid angiopathy; HHcy: Hyperhomocysteinemia; IL: Interleukin; MMP: matrix metalloproteinase; PS1: Presenilin 1; VaD: Vascular dementia.

## Competing interests

The authors declare they have no competing interests.

## Authors’ contributions

TLS performed the experiments including diet administration. EMM and HMB both performed the histological analyses and assisted in manuscript preparation. KB assisted with animal husbandry and genetic analyses. DMW designed the studies, analyzed the data and prepared the manuscript. All authors read and approved the final version of the manuscript.

## References

[B1] Gorelick PB Scuteri A Black SE DeCarli C Greenberg SM Iadecola C Launer LJ Laurent S Lopez OL Nyenhuis D Petersen RC Schneider JA Tzourio C Arnett DK Bennett DA Chui HC Higashida RT Lindquist R Nilsson PM Roman GC Sellke FW Seshadri S on behalf of the American Heart Association Stroke Council, Council on Epidemiology and Prevention, Council on Cardiovascular Nursing, Council on Cardiovascular Radiology and Intervention, and Council on Cardiovascular Surgery and Anesthesia Vascular contributions to cognitive impairment and dementia: a statement for healthcare professionals from the American Heart Association/American Stroke Association Stroke 2011 42 2672 2713 21778438 10.1161/STR.0b013e3182299496 PMC3778669

[B2] Bowler JV Munoz DG Merskey H Hachinski V Fallacies in the pathological confirmation of the diagnosis of Alzheimer’s disease J Neurol Neurosurg Psychiatry 1998 64 18 24 9436722 10.1136/jnnp.64.1.18 PMC2169908

[B3] Zekry D Hauw JJ Gold G Mixed dementia: epidemiology, diagnosis, and treatment J Am Geriatr Soc 2002 50 1431 1438 12165002 10.1046/j.1532-5415.2002.50367.x

[B4] Langa KM Foster NL Larson EB Mixed dementia: emerging concepts and therapeutic implications JAMA 2004 292 2901 2908 15598922 10.1001/jama.292.23.2901

[B5] Sudduth TL Powell DK Smith CD Greenstein A Wilcock DM Induction of hyperhomocysteinemia models vascular dementia by induction of cerebral microhemorrhages and neuroinflammation J Cereb Blood Flow Metab 2013 33 708 715 23361394 10.1038/jcbfm.2013.1 PMC3652696

[B6] Troen AM The central nervous system in animal models of hyperhomocysteinemia Prog Neuropsychopharmacol Biol Psychiatry 2005 29 1140 1151 16111797 10.1016/j.pnpbp.2005.06.025

[B7] Abraham JM Cho L The homocysteine hypothesis: still relevant to the prevention and treatment of cardiovascular disease? Cleve Clin J Med 2010 77 911 918 21147945 10.3949/ccjm.77a.10036

[B8] Troen AM Shea-Budgell M Shukitt-Hale B Smith DE Selhub J Rosenberg IH B-vitamin deficiency causes hyperhomocysteinemia and vascular cognitive impairment in mice Proc Natl Acad Sci U S A 2008 105 12474 12479 18711131 10.1073/pnas.0805350105 PMC2517600

[B9] Pirchl M Ullrich C Humpel C Differential effects of short- and long-term hyperhomocysteinaemia on cholinergic neurons, spatial memory and microbleedings *in vivo* in rats Eur J Neurosci 2010 32 1516 1527 21044172 10.1111/j.1460-9568.2010.07434.x

[B10] Bernardo A McCord M Troen AM Allison JD McDonald MP Impaired spatial memory in APP-overexpressing mice on a homocysteinemia-inducing diet Neurobiol Aging 2007 28 1195 1205 16837103 10.1016/j.neurobiolaging.2006.05.035

[B11] Zhuo JM Praticò D Severe In vivo hyper-homocysteinemia is not associated with elevation of amyloid-β peptides in the Tg2576 mice J Alzheimers Dis 2010 21 133 140 20555139 10.3233/JAD-2010-100171 PMC3880572

[B12] Pacheco-Quinto J Rodriguez de Turco EB DeRosa S Howard A Cruz-Sanchez F Sambamurti K Refolo L Petanceska S Pappolla MA Hyperhomocysteinemic Alzheimer’s mouse model of amyloidosis shows increased brain amyloid β peptide levels Neurobiol Dis 2006 22 651 656 16516482 10.1016/j.nbd.2006.01.005

[B13] Zhuo JM Praticò D Acceleration of brain amyloidosis in an Alzheimer’s disease mouse model by a folate, vitamin B6 and B12-deficient diet Exp Gerontol 2010 45 195 201 20005283 10.1016/j.exger.2009.12.005 PMC2826592

[B14] Jankowsky JL Slunt HH Ratovitski T Jenkins NA Copeland NG Borchelt DR Co-expression of multiple transgenes in mouse CNS: a comparison of strategies Biomol Eng 2001 17 157 165 11337275 10.1016/s1389-0344(01)00067-3

[B15] Alamed J Wilcock DM Diamond DM Gordon MN Morgan D Two-day radial-arm water maze learning and memory task; robust resolution of amyloid-related memory deficits in transgenic mice Nat Protoc 2006 1 1671 1679 17487150 10.1038/nprot.2006.275

[B16] Wilcock DM Lewis MR Van Nostrand WE Davis J Previti ML Gharkholonarehe N Vitek MP Colton CA Progression of amyloid pathology to Alzheimer’s disease pathology in an amyloid precursor protein transgenic mouse model by removal of nitric oxide synthase 2 J Neurosci 2008 28 1537 1545 18272675 10.1523/JNEUROSCI.5066-07.2008 PMC2621082

[B17] Wilcock DM Rojiani A Rosenthal A Subbarao S Freeman MJ Gordon MN Morgan D Passive immunotherapy against Aβ in aged APP-transgenic mice reverses cognitive deficits and depletes parenchymal amyloid deposits in spite of increased vascular amyloid and microhemorrhage J Neuroinflammation 2004 1 24 15588287 10.1186/1742-2094-1-24 PMC539292

[B18] Wilcock DM Zhao Q Morgan D Gordon MN Everhart A Wilson JG Lee JE Colton CA Diverse inflammatory responses in transgenic mouse models of Alzheimer’s disease and the effect of immunotherapy on these responses ASN Neuro 2011 3 249 258 21995345 10.1042/AN20110018 PMC3227004

[B19] Livak KJ Schmittgen TD Analysis of relative gene expression data using real-time quantitative PCR and the 2^−ΔΔ*C*^_T_ method Methods 2001 25 402 408 11846609 10.1006/meth.2001.1262

[B20] Ernest S Hosack A O’Brien WE Rosenblatt DS Nadeau JH Homocysteine levels in A/J and C57BL/6J mice: genetic, diet, gender, and parental effects Physiol Genomics 2005 21 404 410 15741506 10.1152/physiolgenomics.00199.2004

[B21] Wilcock DM Gordon MN Morgan D Quantification of cerebral amyloid angiopathy and parenchymal amyloid plaques with Congo red histochemical stain Nat Protoc 2006 1 1591 1595 17406451 10.1038/nprot.2006.277

[B22] Mantovani A Sica A Sozzani S Allavena P Vecchi A Locati M The chemokine system in diverse forms of macrophage activation and polarization Trends Immunol 2004 25 677 686 15530839 10.1016/j.it.2004.09.015

[B23] Wilcock DM A Changing perspective on the role of neuroinflammation in Alzheimer’s disease Int J Alzheimers Dis 2012 2012 495243 22844636 10.1155/2012/495243 PMC3403314

[B24] Pyo R Lee JK Shipley JM Curci JA Mao D Ziporin SJ Ennis TL Shapiro SD Senior RM Thompson RW Targeted gene disruption of matrix metalloproteinase-9 (gelatinase B) suppresses development of experimental abdominal aortic aneurysms J Clin Invest 2000 105 1641 1649 10841523 10.1172/JCI8931 PMC300851

[B25] Klein T Bischoff R Physiology and pathophysiology of matrix metalloproteases Amino Acids 2011 41 271 290 20640864 10.1007/s00726-010-0689-x PMC3102199

[B26] Wilcock DM Morgan D Gordon MN Taylor TL Ridnour LA Wink DA Colton CA Activation of matrix metalloproteinases following anti-Aβ immunotherapy; implications for microhemorrhage occurrence J Neuroinflammation 2011 8 115 21906275 10.1186/1742-2094-8-115 PMC3182918

[B27] Lo RY Jagust WJ Alzheimer’s Disease Neuroimaging Initiative Vascular burden and Alzheimer disease pathologic progression Neurology 2012 79 1349 1355 22972646 10.1212/WNL.0b013e31826c1b9d PMC3448744

[B28] Nelson PT Abner EL Schmitt FA Kryscio RJ Jicha GA Smith CD Davis DG Poduska JW Patel E Mendiondo MS Markesbery WR Modeling the association between 43 different clinical and pathological variables and the severity of cognitive impairment in a large autopsy cohort of elderly persons Brain Pathol 2010 20 66 79 19021630 10.1111/j.1750-3639.2008.00244.x PMC2864342

[B29] Reed BR Mungas DM Kramer JH Ellis W Vinters HV Zarow C Jagust WJ Chui HC Profiles of neuropsychological impairment in autopsy-defined Alzheimer’s disease and cerebrovascular disease Brain 2007 130 731 739 17267522 10.1093/brain/awl385

[B30] Wilcock DM Alamed J Gottschall PE Grimm J Rosenthal A Pons J Ronan V Symmonds K Gordon MN Morgan D Deglycosylated anti-amyloid-β antibodies eliminate cognitive deficits and reduce parenchymal amyloid with minimal vascular consequences in aged amyloid precursor protein transgenic mice J Neurosci 2006 26 5340 5346 16707786 10.1523/JNEUROSCI.0695-06.2006 PMC6675288

[B31] Wall RT Harlan JM Harker LA Striker GE Homocysteine-induced endothelial cell injury in vitro: a model for the study of vascular injury Thromb Res 1980 18 113 121 7404495 10.1016/0049-3848(80)90175-9

[B32] Chiang JK Sung ML Yu HR Chang HI Kuo HC Tsai TC Yen CK Chen CN Homocysteine induces smooth muscle cell proliferation through differential regulation of cyclins A and D1 expression J Cell Physiol 2011 226 1017 1026 20857402 10.1002/jcp.22415

[B33] Zhang D Xie X Chen Y Hammock BD Kong W Zhu Y Homocysteine upregulates soluble epoxide hydrolase in vascular endothelium in vitro and in vivo Circ Res 2012 110 808 817 22354938 10.1161/CIRCRESAHA.111.259325 PMC3514454

[B34] Hawkes CA Härtig W Kacza J Schliebs R Weller RO Nicoll JA Carare RO Perivascular drainage of solutes is impaired in the ageing mouse brain and in the presence of cerebral amyloid angiopathy Acta Neuropathol 2011 121 431 443 21259015 10.1007/s00401-011-0801-7

[B35] Deane R Yan SD Submamaryan RK LaRue B Jovanovic S Hogg E Welch D Manness L Lin C Yu J Zhu H Ghiso J Frangione B Stern A Schmidt AM Armstrong DL Arnold B Liliensiek B Nawroth P Hofman F Kindy M Stern D Zlokovic B RAGE mediates amyloid-β peptide transport across the blood–brain barrier and accumulation in brain Nat Med 2003 9 907 913 12808450 10.1038/nm890

[B36] Deane R Wu Z Sagare A Davis J Du Yan S Hamm K Xu F Parisi M LaRue B Hu HW Spijkers P Guo H Song X Lenting PJ Van Nostrand WE Zlokovic BV LRP/amyloid β-peptide interaction mediates differential brain efflux of Aβ isoforms Neuron 2004 43 333 344 15294142 10.1016/j.neuron.2004.07.017

[B37] Hofmann MA Lalla E Lu Y Gleason MR Wolf BM Tanji N Ferran LJ Jr Kohl B Rao V Kisiel W Stern DM Schmidt AM Hyperhomocysteinemia enhances vascular inflammation and accelerates atherosclerosis in a murine model J Clin Invest 2001 107 675 683 11254667 10.1172/JCI10588 PMC208940

[B38] Candelario-Jalil E Thompson J Taheri S Grossetete M Adair JC Edmonds E Prestopnik J Wills J Rosenberg GA Matrix metalloproteinases are associated with increased blood–brain barrier opening in vascular cognitive impairment Stroke 2011 42 1345 1350 21454822 10.1161/STROKEAHA.110.600825 PMC3119779

[B39] Lee JM Yin K Hsin I Chen S Fryer JD Holtzman DM Hsu CY Xu J Matrix metalloproteinase-9 in cerebral-amyloid-angiopathy-related hemorrhage J Neurol Sci 2005 229–230 249 254 10.1016/j.jns.2004.11.041 15760647

[B40] Hernandez-Guillamon M Martinez-Saez E Delgado P Domingues-Montanari S Boada C Penalba A Boada M Pagola J Maisterra O Rodriguez-Luna D Molina CA Rovira A Alvarez-Sabin J Ortega-Aznar A Montaner J MMP-2/MMP-9 plasma level and brain expression in cerebral amyloid angiopathy-associated hemorrhagic stroke Brain Pathol 2012 22 133 141 21707819 10.1111/j.1750-3639.2011.00512.x PMC8029059

